# Modeling the activity burst in the initial phase of cellulose hydrolysis by the processive cellobiohydrolase Cel7A

**DOI:** 10.1002/bit.26889

**Published:** 2019-01-08

**Authors:** Zdeneˇk Petrášek, Manuel Eibinger, Bernd Nidetzky

**Affiliations:** ^1^ Institute of Biotechnology and Biochemical Engineering Graz University of Technology, NAWI Graz Graz Austria; ^2^ Austrian Centre of Industrial Biotechnology Graz Austria

**Keywords:** activity burst, cellobiohydrolase, cellulose, hydrolysis, modeling, processive action

## Abstract

The hydrolysis of cellulose by processive cellulases, such as exocellulase *Tr*Cel7A from *Trichoderma reesei*, is typically characterized by an initial burst of high activity followed by a slowdown, often leading to incomplete hydrolysis of the substrate. The origins of these limitations to cellulose hydrolysis are not yet fully understood. Here, we propose a new model for the initial phase of cellulose hydrolysis by processive cellulases, incorporating a bound but inactive enzyme state. The model, based on ordinary differential equations, accurately reproduces the activity burst and the subsequent slowdown of the cellulose hydrolysis and describes the experimental data equally well or better than the previously suggested model. We also derive steady‐state expressions that can be used to describe the pseudo‐steady state reached after the initial activity burst. Importantly, we show that the new model predicts the existence of an optimal enzyme‐substrate affinity at which the pseudo‐steady state hydrolysis rate is maximized. The model further allows the calculation of glucose production rate from the first cut in the processive run and reproduces the second activity burst commonly observed upon new enzyme addition. These results are expected to be applicable also to other processive enzymes.

## 1 INTRODUCTION

The biological degradation of biopolymers, such as polysaccharides, proteins, and nucleic acids involves processively acting hydrolases. Processivity means that after binding the substrate, the enzyme remains attached to it and performs multiple rounds of catalysis before dissociating again (Breyer & Matthews, 2001). Processive hydrolysis is particularly important in enzymatic degradation of cellulose (Payne et al., 2015). The cellobiohydrolase *Tr*Cel7A from *Trichoderma reesei* is the main component of industrial cellulase systems and is essential for the degradation of crystalline cellulose (Nakamura et al., 2014; Payne et al., 2015; Praestgaard et al., 2011).

An important role in deciphering the mechanism of cellulose hydrolysis is played by mathematical modeling (Bansal, Hall, Realff, Lee, & Bommarius, 2009; Jeoh, Cardona, Karuna, Mudinoor, & Nill, 2017), including both the models based on ordinary differential equations (Bommarius et al., 2008; Griggs, Stickel, & Lischeske, 2012; Praestgaard et al., 2011) and on particle‐based simulations (Eibinger, Zahel, Ganner, Plank, & Nidetzky, 2016; Shang, Chang, & Chu, 2013; Shang & Chu, 2014; Warden, Little, & Haritos, 2011).

A key obstacle for an efficient utilization of lignocellulosic biomass is the substrate recalcitrance, manifested by, among other effects, a rapid slowdown of the enzymatic deconstruction of cellulose within minutes after the initial activity burst phase at low cellulose conversion (Cruys‐Bagger et al., 2012; Murphy et al., 2012; Praestgaard et al., 2011). The origin of the activity burst has been attributed to either the heterogeneous nature of the substrate and the changes of the substrate morphology in the course of hydrolysis (Beckham et al., 2011; Ganner et al., 2012; Grethlein, 1985; Peciulyte, Karlström, Larsson, & Olsson, 2015), or to the enzyme‐related phenomena, such as inactivation of cellulases due to irreversible nonproductive binding onto the cellulose (Cruys‐Bagger et al., 2012, 2016; Kostylev & Wilson, 2013; Kurašin, Kuusk, Kuusk, Sørlie & Väljamäe, 2015). Although experimental evidence from studies employing either pre‐steady‐state measurements (Cruys‐Bagger et al., 2012; Olsen, Kari, Borch & Westh, 2017) or time‐resolved high‐resolution visualization techniques (Igarashi et al., 2011; Nakamura et al., 2014) provided significant insights regarding the quantitative understanding of cellobiohydrolase action, many fundamental questions still remain open.

The burst kinetics observed in the first‐cut product release in cellulose hydrolysis (Kipper, Väljamäe & Johansson, 2005) has led to mechanistic models of the hydrolysis burst based on the idea of the enzyme becoming reversibly blocked while processively hydrolyzing the cellulose chain (obstacle model; Jalak & Väljamäe, 2010). Praestgaard et al. (2011) published an analytical model for the initial burst phase of processive cellulose hydrolysis employing the obstacle concept and used it to describe the experimentally observed hydrolysis of amorphous cellulose by the cellobiohydrolase Cel7A. The model has been applied in analysis of cellulose hydrolysis in several subsequent studies (Cruys‐Bagger et al., 2012; Cruys‐Bagger, Elmerdahl, Praestgaard, Borch, & Westh, 2013; Cruys‐Bagger, Tatsumi, Ren, Borch, & Westh, 2013; Kari et al., 2014; Sørensen et al., 2015, 2017).

**Figure 1:**
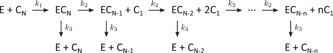
The model of processive cellulose hydrolysis published in Praestgaard et al. (2011). Symbols: E—free (not bound) enzyme, $C_{\text{N}}$—cellulose chain of N cellobiose units, ${\text{EC}}_{\text{N}}$—enyzme complexed to the cellulose chain of N units, ${\text{C}}_{1}$—cellobiose

The model (Figure 1) assumes that the cellulase molecule attaches to the cellulose chain (rate constant $k_{1}$) and processively hydrolyzes the cellulose (rate constant $k_{2}$) with the possibility of detachment from the cellulose at any time (rate constant $k_{3}$). After performing at most n hydrolysis steps the enzyme detaches (rate constant $k_{3}$).

The solution of the rate equations derived from the scheme in Figure 1 was used to fit the model to the experimental data by adjusting the four parameters, $k_{1}$, $k_{2}$, $k_{3}$, and n (Praestgaard et al., 2011). Although the model predicts an activity burst shortly after mixing the enzyme with the substrate, the fits to the data are rather poor (Figure 5a and Supplementary Information Figure S2). The two main shortcomings of the model, as also stated in the original publication (Praestgaard et al., 2011), are the abrupt and steep decrease of activity at the end of the activity burst, and the fast convergence to the steady state (constant hydrolysis rate) following the activity burst. In contrast to the experimental data, the time when the maximum of the activity is reached in the model is only weakly dependent on the substrate concentration.

A close inspection of the published model suggests that the main reason for the deficiency of this model can be linked to the fixed maximum number of hydrolysis steps n that the enzyme is allowed to perform before it must detach from the cellulose chain. This can be seen by estimating the time the enzyme needs to perform n hydrolysis steps using the values of n=150 and $k_{2}=0.55\;s^{-1}$ from the fit: the time is $n∕k_{2}\approx 270$ s, which coincides with the time of the abrupt activity decrease in the model (Supplementary Information Figure S2). To improve the theoretical description of the data, the authors modified the model to include several values of n instead of only one (Praestgaard et al., 2011). This, however, did not lead to a significantly better match of the model with the data. The second shortcoming of the published model, a fast convergence to the steady state, could be eliminated only by including an additional assumption of irreversible enzyme inactivation in course of time.

Here we propose a minimal model for the initial phase of cellulose hydrolysis by *Tr*Cel7A inspired by earlier experiments and a pre‐steady‐state model by Praestgaard et al. (2011). The model accurately reproduces the initial hydrolysis burst observed before a significant fraction of substrate is converted, while remaining as simple as possible, in terms of the number of kinetic parameters, distinct reaction species, and reactions. The simple analytical solution of the model greatly facilitates the analysis of experimental data. Furthermore, we derive the expressions describing the steady‐state of the presented model, and show that the model provides important insights into the kinetics of processive cellulose hydrolysis.

## 2 COMPUTATIONAL METHODS

The systems of ordinary differential equations (Equation (3)) were solved in Matlab (MathWorks, Natick, MA) by evaluating the eigenvalues and eigenvectors of the matrix A using the Matlab function eig(), as described in Section 3. The published data (Praestgaard et al., 2011) were fitted to the presented models using the Matlab function lsqnonlin() which uses the standard least‐squares minimization algorithm to find the optimal values of the unknown parameters by minimizing the difference between the data and the model.

## 3 MODEL CONSTRUCTION

### 3.1 Proposed model

As shown below, the weaknesses of the published model were eliminated and a better agreement with the data was achieved by removing the limit of maximum number n of hydrolysis steps that are possible, and introducing an additional, transiently inactive, enzyme state, which is effectively playing the role of the enzyme state EC_*N‐n*_ in the published model (Figure 1).

Since we are interested in describing the activity burst during the initial phase of the cellulose hydrolysis, we make the following assumptions (identical with those in Praestgaard et al., 2011). First, the substrate concentration $c_{0}$ does not change during the hydrolysis. This follows from the observed low overall conversion during the initial hydrolysis phase. Second, the enzyme‐binding sites on the substrate are in excess of the enzyme. The consequence of this assumption is that instead of the bimolecular binding rate constant $k_{1}^{\prime }$ one can use a unimolecular rate constant $k_{1}=k_{1}^{\prime }c_{0}$, independent of the concentration of the bound enzyme species. This assumption has been confirmed by experiments with varying enzyme concentration and constant substrate concentration (Praestgaard et al., 2011). Third, we neglect any long‐time effects, such as permanent enzyme deactivation. This allows us to keep the model simple, but it also means that the model cannot be expected to describe the commonly observed slowdown of hydrolysis at very long times.

**Figure 2:**
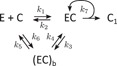
The proposed model of processive cellulose hydrolysis with three enzyme states. Symbols: E—free enzyme, EC—active bound enzyme, ${\left(\text{EC}\right)}_{\text{b}}$—inactive bound enzyme, C—cellulose chain, and ${\text{C}}_{1}$—cellobiose

In the proposed model (Figure 2), the initially free enzyme binds to the cellulose chain and is present either in an active state EC or in an inactive (blocked) state ${\left(\text{EC}\right)}_{\text{b}}$. The enzyme in the active state is hydrolyzing cellulose with the rate constant $k_{7}$ without being released from cellulose upon hydrolysis. The complete model allows interconversion between all three enzyme states (E: free in solution, EC: active bound, ${\left(\text{EC}\right)}_{\text{b}}$: inactive bound), described by the rate constants $k_{1}$–$k_{6}$.

We introduce the following notation: $y_{1}$ is the concentration of the free enzyme in solution E, $y_{2}$ is the concentration of the bound, active enzyme EC, $y_{3}$ is the concentration of the bound, inactive enzyme ${\left(\text{EC}\right)}_{\text{b}}$, and $y_{T}$ is the total enzyme concentration: $y_{T}=y_{1}+y_{2}+y_{3}$.

The reaction scheme in Figure 2 leads directly to the following rate equations:

(1) $$\begin{array}{ccc} {\dot{y}}_{1} & = & \left(-k_{1}-k_{6}\right)y_{1}+k_{2}y_{2}+k_{5}y_{3},\\ {\dot{y}}_{2} & = & +k_{1}y_{1}+\left(-k_{2}-k_{3}\right)y_{2}+k_{4}y_{3},\\ {\dot{y}}_{3} & = & +k_{6}y_{1}+k_{3}y_{2}+\left(-k_{4}-k_{5}\right)y_{3}. \end{array}$$

Since the total amount of enzyme $y_{T}$ is conserved, we can simplify the equations by substituting for $y_{3}:y_{3}=y_{T}-y_{1}-y_{2}$, which leads to

(2) $$\begin{array}{ccc} {\dot{y}}_{1} & = & k_{5}y_{T}-\left(k_{1}+k_{6}+k_{5}\right)y_{1}+\left(k_{2}-k_{5}\right)y_{2},\\ {\dot{y}}_{2} & = & k_{4}y_{T}+\left(k_{1}-k_{4}\right)y_{1}-\left(k_{2}+k_{3}+k_{4}\right)y_{2}. \end{array}$$

This set of equations can be written using a matrix form:

(3) $$\dot{y}=b+\mathrm{Ay},$$

where the vectors b and y and the matrix A are defined as

(4) $$b=\left(\begin{array}{c} k_{5}y_{T}\\ k_{4}y_{T} \end{array}\right),\;A=\left(\begin{array}{cc} -\;k_{1}-k_{6}-k_{5} & +\;k_{2}-k_{5}\\ +\;k_{1}-k_{4} & -\;k_{2}-k_{3}-k_{4} \end{array}\right),\;y=\left(\begin{array}{c} y_{1}\\ y_{2} \end{array}\right).$$

The linear ordinary differential equation (3) has the solution:

(5) $$y=Ve^{Dt}\;V^{-1}\left(y_{0}-y_{\text{ss}}\right)+y_{\text{ss}},$$

where V is the matrix of eigenvectors of A (ordered columnwise), D is a diagonal matrix with the eigenvalues of A on the diagonal, $V^{-1}$ is the inverse matrix of V, $y_{0}$ are the initial concentrations of the enzyme species (we assume that initially all the enzyme is in the state E), and $y_{\text{ss}}$ are the steady‐state concentration values:

(6) $$y_{0}=\left(\begin{array}{c} y_{T}\\ 0 \end{array}\right),\;y_{\text{ss}}=-A^{-1}b.$$

The solution given by Equation (5) is analytical; the concentrations are sums of two exponential terms plus a constant value (the steady‐state value). The eigenvalues and eigenvectors of A can be directly calculated in Matlab or a similar software. Equation (5) can be therefore conveniently used to fit the experimental data.

The time‐dependent rate of hydrolysis ${\dot{c}}_{p}\left(t\right)$, which can be directly compared with the experimental data, is calculated from the solution of the model as ${\dot{c}}_{p}\left(t\right)=k_{7}y_{2}\left(t\right)$. It should be stressed that the solution $y_{i}\left(t\right)$ is independent of the hydrolysis rate constant $k_{7}$, since it is determined only by the transitions between the three enzyme states which are not coupled to the hydrolysis reaction. A direct consequence is that the solution covers also the situations when the hydrolysis is very slow compared to the time the enzyme stays in the active state, that is, even when the enzyme processivity is negligible.

**Figure 3:**
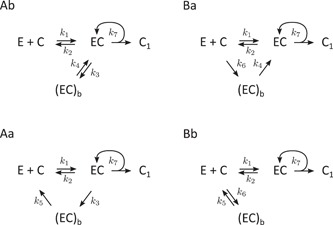
The four‐rate models that produce good fits to the experimental data (Figure 5). The models are derived from the full model (Figure 2) by keeping only four of the six reactions interconverting the three enzyme species. The models form two pairs: (Ab, Ba) and (Aa, Bb); the models within each pair are equivalent from the point of view of available data, that is, they produce equal fits and cannot be distinguished from each other

The full model has seven parameters: $k_{1}\ldots k_{7}$. This is a rather large number, and it can hardly be expected that the fits to the data can uniquely determine all seven parameters. Therefore, simplified variations of this model with the dominant reactions retained and the less significant reactions removed (the corresponding rates were set to zero) were considered. As described in detail in Supplementary Information, models containing four of the six possible reactions interconverting the enzyme species (the “four‐rate” models) represent the minimal models for the description of the data considered in this study. These models contain five kinetic parameters: the four interconversion rate constants (out of the six: $k_{1}$–$k_{6}$) and the hydrolysis rate constant $k_{7}$. Of these, four models, denoted Ab, Aa, Ba, and Bb (Figure 3), yielded the best fits to the data, as described in the following section.

Because of the limited experimental data—in this case, a set of time‐dependent hydrolysis rates determined for several substrate concentrations—it is not always possible to distinguish between different models because they lead to exactly the same predictions, only with modified parameters and scaling factors. We call such models equivalent and describe their relationships and transformations in more detail in the Supplementary Information. It turns out that the models in Figure 3 form two groups of equivalent models: the model Ab is equivalent to Ba, and the model Aa is equivalent to Bb. This means, for example, that parameters of the fitted Ab model ($k_{1},k_{2},k_{3},k_{4},k_{7}$) can be transformed to the parameters of the Ba model ($k_{1},k_{2},k_{4},k_{6},k_{7}$) producing the same fit, that is, the same time evolution of the hydrolysis rate.

### 3.2 Steady state

The proposed model implies that after the initial period the system reaches a steady state. The dependence of the concentration of the hydrolyzing enzyme species EC in the steady state ($y_{2ss}$) on the substrate concentration $c_{0}$ (which is implicitly included in the unimolecular rate constant $k_{1}=k_{1}^{\prime }c_{0}$) has the usual hyperbolic form:

model Aa:

(7) $$y_{2ss}=y_{T}\frac{k_{5}}{k_{3}+k_{5}}\frac{k_{1}}{k_{1}+(k_{5}\left(k_{2}+k_{3}\right))/(k_{3}+k_{5})},$$

model Ab:

(8) $$y_{2ss}=y_{T}\frac{k_{4}}{k_{3}+k_{4}}\frac{k_{1}}{k_{1}+{(k}_{2}k_{4}/(k_{3}+k_{4}))}.$$

The steady‐state rate of the processive hydrolysis $v_{ss}$ is directly proportional to the steady‐state concentration of the active species EC: $v_{ss}=k_{7}y_{2ss}$. This allows Equations (7) and (8) to be written in a form analogous to the Michaelis–Menten formula with modified parameters:

(9) $$v_{ss}=v_{\text{max}}^{\prime }\frac{c_{0}}{c_{0}+K_{M}^{\prime }}$$

with the following effective maximum reaction rate $v_{\text{max}}^{\prime }$ and the effective Michaelis constant $K_{M}^{\prime }$ for the model Aa:

(10) $$v_{\text{max}}^{\prime }=k_{7}y_{T}\frac{k_{5}}{\left(k_{3}+k_{5}\right)},\;K_{M}^{\prime }=\frac{k_{5}\left(k_{2}+k_{3}\right)}{k_{1}^{\prime }\left(k_{3}+k_{5}\right)}$$

and for the model Ab:

(11) $$v_{\text{max}}^{\prime }=k_{7}y_{T}\frac{k_{4}}{\left(k_{3}+k_{4}\right)},\;K_{M}^{\prime }=\frac{k_{4}k_{2}}{k_{1}^{\prime }\left(k_{3}+k_{4}\right)}.$$

Compared to the ideal case (Michaelis–Menten), the maximum hydrolysis rate $v_{\text{max}}^{\prime }$ is limited by the ratios $k_{5}∕\left(k_{3}+k_{5}\right)$ and $k_{4}∕\left(k_{3}+k_{4}\right)$ in the two models (Aa and Ab). These ratios are always lower than or equal to one, and represent the maximum fractions of the enzyme in the active state EC (reached in the limit of very high substrate concentration). In other words, they reduce the ideal maximal hydrolysis rate by the fraction of the enzyme in the blocked state ${\left(\text{EC}\right)}_{\text{b}}$.

A steady‐state theory based on the published reaction scheme shown in Figure 1 has been formulated previously (Cruys‐Bagger, Elmerdahl, et al., 2013). There, the maximum hydrolysis rate is given by a formula analogous to Equations (10) and (11), with a limiting factor called ‘kinetic processivity coefficient’ *β*, a fraction expressing the probability that the bound enzyme is not at the end of its n hydrolysis steps, in the EC_*N‐n*_ state (blocked, waiting to dissociate). In other words, *β* is the probability, that the enzyme is actively hydrolyzing the cellulose chain, analogously to the limiting ratio in Equations (10) and (11). From this point of view, the steady‐state descriptions of the published and suggested models are directly comparable.

### 3.3 Glucose production

**Figure 4:**
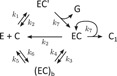
The proposed model of processive cellulose hydrolysis incorporating the assumption that the first hydrolysis cut in the processive run (EC′ → EC) produces glucose instead of cellobiose. The EC′ is the active enzyme state before performing any hydrolysis step, EC is the active enzyme after performing at least one hydrolysis step, G stands for glucose; other symbols as in Figure 2

The first hydrolysis cut in the processive run after binding of the enzyme to the cellulose and threading the cellulose chain may result in production of a glucose, a cellobiose, or a cellotriose molecule; the subsequent steps are thought to produce only cellobiose (Fox, Levine, Clark, & Blanch, 2012; Kari et al., 2017). The rate of glucose production in the first cut can be modeled within the proposed reaction scheme by separating the active state EC into two populations: the molecules that are bound but have not performed any hydrolysis yet (EC′), and the molecules that have hydrolyzed at least one bond (EC), as shown in Figure 4.

This mechanism leads to a set of differential equations in the form of Equation (3) with the following definitions of the vectors b and y and the matrix A, instead of Equation (4):

(12) $$\begin{array}{ccc} b & = & \left(\begin{array}{c} k_{5}y_{T}\\ 0\\ k_{4}y_{T} \end{array}\right),\;A=\left(\begin{array}{ccc} -k_{1}-k_{6}-k_{5}, & +k_{2}-k_{5}, & +k_{2}-k_{5}\\ +k_{1}, & -k_{2}-k_{7}, & 0\\ -k_{4}, & +k_{7}-k_{4}, & -k_{2}-k_{3}-k_{4} \end{array}\right),\\ y & = & \left(\begin{array}{c} y_{1}\\ y_{2}^{\prime }\\ y_{2} \end{array}\right), \end{array}$$

where $y_{2}^{\prime }$ is the concentration of the bound, active enzyme EC′ that has not performed any hydrolysis step yet, and $y_{2}$ is the concentration of the bound, active enzyme EC that has performed at least one hydrolysis step. The solution is given by Equations (5) and (12).

## 4 RESULTS AND DISCUSSION

### 4.1 Hydrolysis burst

**Figure 5:**
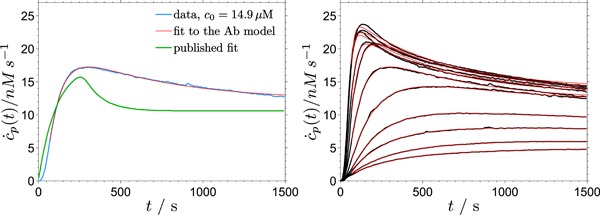
The fits of the published cellulose hydrolysis data. Left: the experimental temporal change of the rate of cellobiose production (initial substrate concentration $c_{0}=14.9\;\mu$M) together with a fit to the Ab model and a published fit (published data and fits from Praestgaard et al. (2011). Right: the published cellobiose production rates and the corresponding global fit to the Ab model. The data with the initial substrate concentrations in the range from 1.5 to 110.9 μM are shown. The cellobiose production rate in the fits is calculated as ${\dot{c}}_{p}\left(t\right)=k_{7}y_{2}\left(t\right)$ [Color figure can be viewed at wileyonlinelibrary.com]

The time‐dependent rate of cellobiose production by *Tr*Cel7A shows strong variations during the initial hydrolysis phase, exhibiting a distinct activity burst at high substrate concentrations (Cruys‐Bagger et al., 2012; Praestgaard et al., 2011). We have analyzed the published experimental data (Figure 5) using the four‐rate models presented in Figure 3, the full model (Figure 2), and the other related models discussed in the Supplementary Information. The analysis was performed globally, meaning that all data sets were fitted simultaneously and all the common fitting parameters (rate constants) were forced to be the same for all substrate concentrations $c_{0}$, with the exception of the unimolecular rate constant $k_{1}=k_{1}^{\prime }c_{0}$, which naturally varies between experiments with different substrate concentration $c_{0}$. The total enzyme concentration $y_{T}$, needed to determine the hydrolysis rate constant $k_{7}$, was 50 nM.

The best fits were obtained with the models Ab (or, equivalently, Ba) and Aa (Bb), with no significant difference between the two. The parameters obtained from the fits, compared to the parameters of the published model (Praestgaard et al., 2011), are listed in Table 1. The fits using the other four‐rate models (Supporting Information Figure S1) were of lower quality. The fit with the full model (Figure 2) was not appreciably better than the fits to the models Ab/Ba or Aa/Bb. The values of the fit parameters were, however, very close to those of model Ab. This shows that given the available data the six interconversion rates are redundant, and the reduced models with four rates are sufficient to describe the data.

**Table 1:** The parameters obtained from the fits of the models Ab, Ba, Aa, and Bb to the data in Figure 5

Parameters	Units	Ab	Ba	Aa	Bb	Published
$k_{1}^{\prime }=k_{1}∕c_{0}$	$s^{-1}\;\mu M^{-1}$	0.00063	0.00040	0.00064	0.00043	0.0004 ($k_{1}$)
$k_{2}$	$s^{-1}$	0.0020	0.0032	0.0027	0.0035	0.0034 ($k_{3}$)
$k_{3}$	$s^{-1}$	0.00081	0	0.00081	0	
$k_{4}$	$s^{-1}$	0.0010	0.00063	0	0	
$k_{5}$	$s^{-1}$	0	0	0.0010	0.0010	0.0034 ($k_{3}$)
$k_{6}$	$s^{-1}\;\mu M^{-1}$	0	0.59$k_{1}$	0	0.48$k_{1}$	
$k_{7}$	$s^{-1}$	0.49	0.78	0.57^a^	0.84	0.55 ($k_{2}$)
$t_{p}$	s	357	314	286	370	177
$\bar{n}=t_{p}k_{7}$	1	175	245	163^a^	311	98 (n=150)
$p_{b}$	–	0.29	0	0.23	0	0.40
$y_{2ss}∕y_{T},c_{0}\to \infty$	–	0.57	0.36	0.65	0.44	~0.6

*Note.* Explanation of the additional parameters: $t_{p}$ is the average duration of one processive run: $t_{p}=1∕\left(k_{2}+k_{3}\right)$ in the Ab and Aa models and $t_{p}=1∕k_{2}$ in the Ba and Bb models; $\bar{n}=t_{p}k_{7}$ is the processivity (the average number of hydrolyzed units in one processive run; $\bar{n}=k_{2}∕k_{3}\left(1-{\left(k_{2}∕\left(k_{2}+k_{3}\right)\right)}^{n}\right)$ in the published model; see Supplementary Information); $p_{b}$ is the probability of reaching the blocked state before dissociating; $p_{b}={\left(k_{2}∕\left(k_{2}+k_{3}\right)\right)}^{n}={\left(k_{\text{cat}}∕\left(k_{\text{cat}}+k_{\text{off}}\right)\right)}^{n}$ in the published model; $p_{b}=k_{3}∕\left(k_{2}+k_{3}\right)$ in the proposed models Aa and Ab, and $p_{b}=0$ in the models Ba and Bb; $y_{2ss}∕y_{T}$ is the fraction of the enzyme in the active state (EC) in the limit of infinite substrate concentration ($c_{0}\to \infty$). The symbols in parentheses in the last column are the parameter names used in the corresponding publication (Praestgaard et al., 2011).

^a^ excluding the lowest three substrate concentrations.

Since the equivalent models (Ab/Ba or Aa/Bb) produce equal fits, any preference of one model to the other one in each pair can be made only on the basis of the obtained parameter values. The most significant difference between Type A and Type B models, as can be seen in Table 1, is the average number of hydrolyzed units in one processive run $\bar{n}=t_{p}k_{7}$: while $\bar{n}$ is 175 and 163 for the Ab and Aa models, it is higher for the Ba and Bb models: 245 and 311. The value of $\bar{n}$ is expected to be smaller than the degree of polymerization of the cellulose chain, which has been reported as dp = 210 for the used substrate (Zhang & Lynd, 2005). Based on this reasoning, the models Ab and Aa appear to be preferable to the models Ba or Bb. Furthermore, considering the steady‐state analysis shown below, the model pair Ab/Ba describes the data slightly better than the pair Aa/Bb. This leaves the model Ab as the most preferable description of the data. It should be stressed, however, that the differences between the models are rather small, and a definite conclusion cannot be drawn at this point. To better discriminate between different models, additional independent data should be obtained. This could be, for example, the time‐dependent concentrations of the unbound enzyme E and the enzyme in the blocked state ${\left(\text{EC}\right)}_{\text{b}}$.

Comparison of the fits to this data set using the new model proposed here with the fits using the previously published model (Figure 5, Supporting Information Figure S2) shows a much better fit with the new model. The model matches the data completely in the lower substrate concentration range. There is a small discrepancy between the data and the fit near the maximum of the activity burst at the highest substrate concentrations, which can be attributed to the simplicity of the model, or to the limited temporal resolution of the experimental method. A relevant simplification may be, for example, the assumption of only one type of the inactive bound state ${\left(\text{EC}\right)}_{\text{b}}$, while in reality it is plausible that the hydrolysis by a bound enzyme may not be possible for many reasons, as discussed below.

The reason for the success of the proposed model can be linked to the absence of the fixed chain length n to be hydrolyzed. For the activity burst to appear, the blocked state ${\left(\text{EC}\right)}_{\text{b}}$ is necessary. In the published model (Praestgaard et al., 2011), the state EC_*N‐n*_ plays the role of the blocked state, because the enzyme in this state cannot hydrolyze anymore, and can only detach from the substrate. Because this blocked state is reached after a fixed number n of hydrolysis steps, the activity burst exhibits an abrupt fall‐off. In the proposed model the blocked state can be reached after any hydrolysis step, resulting in a smooth activity burst, without a sharp fall‐off and with a prolonged approach toward the steady state (Supporting Information Figure S2). Interestingly, the slower approach toward the steady state means that the model matches the data well even at longer times, and this without including enzyme inactivation, as was necessary with the published model (Praestgaard et al., 2011).

In the models of Type A (Ab, Aa), the free enzyme binds and enters first the active state EC. The blocked state ${\left(\text{EC}\right)}_{\text{b}}$ is entered only later, which, intuitively, lowers the population of the active state, possibly resulting in the appearance of the activity maximum. In the models of Type B (Ba, Bb), the enzyme binds the substrate to enter either the active or the blocked state. The appearance of the activity maxima is less intuitive here, but may be understood in this way: if the rate of entering the active state $k_{1}$ is higher than the rate of entering the blocked state $k_{6}$, the active state may initially reach a high level, before it is lowered again while the blocked state is being populated and the concentrations of all three enzyme states tend to a steady state.

The difference between the models Ab and Aa is only in the way the blocked state is left: back to the active state on the substrate (Ab) or dissociate from the substrate (Aa). This difference does not seem to be particularly relevant for the overall kinetics, resulting in only small differences in the fits with the two models. The same reasoning applies to the difference between the models Ba and Bb.

The abstract models naturally do not contain any information about the physical nature of the postulated blocked state. In models Aa and Ab, the blocked state may represent a situation when the hydrolyzing enzyme encounters an obstacle, such as another substrate structure, another bound enzyme, or the currently hydrolyzed cellulose chain may lead deeper into the substrate where it cannot be followed by the enzyme anymore. Evidence suggesting such mechanism of temporary blocking the hydrolysis has been presented before (Igarashi et al., 2011; Jalak & Väljamäe, 2010; Kurašin & Väljamäe, 2011; Väljamäe, Sild, Pettersson, & Johansson, 1998; Yang, Willies, & Wyman, 2006). In models Ba and Bb, the blocked state may additionally be any binding state where the enzyme is not hydrolyzing (yet), for example, because it has not threaded a cellulose chain (yet), or because it is searching on the substrate surface for the active site. Explanations of experiments based on this mechanism have appeared in several published works (Fox et al., 2012; Maurer, Bedbrook, & Radke, 2012; Shang et al., 2013).

The application of the proposed model to the data from Praestgaard et al. (2011) yielded kinetic parameters that are comparable to those provided by the published model (Table 1) and agree with some other published data. There are, however, numerous reports of generally higher rate constants and lower processivity values. For example, the detachment rate constant $k_{2}$ of $\sim 0.003\;s^{-1}$ agrees with values published elsewhere (Kont, Kari, Borch, Westh, & Väljamäe, 2016; Kurašin & Väljamäe, 2011), but significantly higher values (0.01–0.02 $s^{-1}$) have also been reported (Cruys‐Bagger et al., 2012; Cruys‐Bagger, Tatsumi, et al., 2013). The hydrolysis rate constant $k_{7}$ ($k_{\text{cat}}$) of $\sim 0.5\;s^{-1}$ is lower than most reported values, reaching up to $\sim 7\;s^{-1}$(Igarashi et al., 2011). The processivity value of $\bar{n}\sim 170$ from the proposed model is somewhat larger than the value of 98 from the model by Praestgaard et al. (2011) but considerably larger than typical experimental processivities, lying in the range of 10–20 (Cruys‐Bagger et al., 2012; Kurašin & Väljamäe, 2011; Nill, Karuna, & Jeoh, 2018). However, it is important to note that the experimentally determined processivity, usually termed the ‘apparent processivity,’ is typically smaller than the ‘intrinsic processivity’ defined on basis of a theoretical model using rate constants. An intrinsic processivity of several hundred has been estimated before Kurašin and Väljamäe (2011).

**Figure 6:**
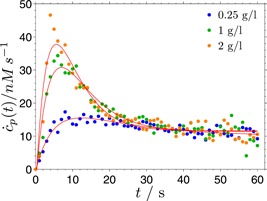
The experimental rate of cellobiose production (points) from Cruys‐Bagger et al. (2012) together with fits (curves) to the proposed model Ab. Three substrate concentration were used: 0.25, 1, and 2 g/L [Color figure can be viewed at wileyonlinelibrary.com]

To see how the proposed model performs on data with a faster initial kinetics, we have also analyzed similar data of the hydrolysis of regenerated amorphous cellulose by Cel7A, but measured with a different technique (Cruys‐Bagger et al., 2012). In this case, the fits by the published model (which are already of good quality) are comparable to the fits by the proposed model (Figure 6). The data set consists of data covering a narrower substrate concentration range, contains less data points and is more noisy than the data analyzed above (Figure 5), making it more difficult to discriminate between alternative models on basis of the fit quality and resulting in larger uncertainty of the fit parameters. The fit parameters of the two models are compared in Table 2. The hydrolysis burst in this data set occurs on considerably shorter time scales than in the data in Figure 5 and Supporting Information Figure S2. Consequently, the hydrolysis rate constant $k_{7}=1.7$–$2.5\;s^{-1}$ is larger, approaching the values determined by other studies (Igarashi et al., 2011). The processivity of ~10 is also closer to the experimentally determined processivity (Kurašin & Väljamäe, 2011).

**Table 2:** The parameters obtained from the fits of the models Ab and Aa to the data in Figure 6

Parameters	Units	Ab	Aa	Published
$k_{1}^{\prime }=k_{1}∕c_{0}$	$s^{-1}{\;g}^{-1}\;l$	0.12	0.097	0.06 ($k_{on}$)
$k_{2}$	$s^{-1}$	$2.6\;\times \;10^{-12}$	0.0074	0.022 ($k_{off}$)
$k_{3}$	$s^{-1}$	0.18	0.19	
$k_{4}$	$s^{-1}$	0.027	0	
$k_{5}$	$s^{-1}$	0	0.028	0.022 ($k_{off}$)
$k_{6}$	$s^{-1}{\;g}^{-1}\;l$	0	0	
$k_{7}$	$s^{-1}$	1.7	2.5	4 ($k_{cat}$)
$t_{p}$	s	5.6	5.0	3.1
$\bar{n}=t_{p}k_{7}$	1	9.5	12.5	12.5 (n=13)
$p_{b}$	–	1.0	0.96	0.93
$y_{2ss}∕y_{T},c_{0}\to \infty$	–	0.13	0.125	~0.1

*Note*: The last column contains the values from the original publication (Cruys‐Bagger et al., 2012). The parameters $t_{p}$, $\bar{n}$, $p_{b}$ and $y_{2ss}∕y_{T}$ are explained in the legend to Table 1.

The two data sets analyzed in this study were obtained using different experimental methods. The overall slower kinetics measured with the calorimetric method could be related to the instrument time constant of 15 s, as opposed to the time constant of the biosensor of 1 s. However, it seems unlikely that this methodological difference alone could provide the full explanation for the observed differences in the hydrolysis kinetics in the two data sets.

### 4.2 Steady state

**Figure 7:**
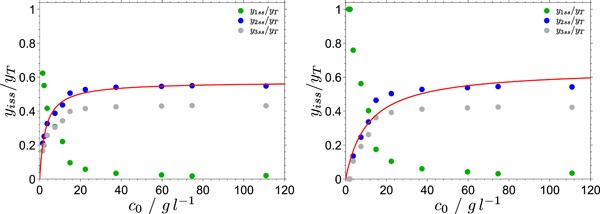
The relative concentrations $y_{iss}∕y_{T}$ of the three enzyme species, E ($y_{1ss}∕y_{T}$), EC ($y_{2ss}∕y_{T}$), and ${\left(\text{EC}\right)}_{\text{b}}$ ($y_{3ss}∕y_{T}$) in the steady state. The relative concentration of the active species EC is fitted to the hyperbolic dependence on the substrate concentration $c_{0}$ (Equations (7) and (8)); left: model Ab and right: model Aa ($y_{T}$ is the total enzyme concentration) [Color figure can be viewed at wileyonlinelibrary.com]

The experimental data suggest that after the initial burst the reaction reaches a pseudo‐steady state, where the hydrolysis rate changes only weakly (Figure 5). The steady‐state concentration of the active species EC can be obtained from the fits shown in Figure 5, and its dependence on the substrate concentration $c_{0}$ can be fitted to a hyperbolic function (Equations (7) and (8)), see Figure 7. Although the fits of the two models (Aa and Ab) to the kinetic data (Figure 5) are of equal quality, the model Ab produces a somewhat better fit to the steady‐state data than model Aa (Figure 7). For this reason, model Ab is slightly more preferred over model Aa.

### 4.3 Substrate affinity, processivity, and hydrolysis rate

It has been suggested that the affinity of the enzyme to the cellulose substrate may have nontrivial influence on the overall hydrolysis rate (Nakamura et al., 2014). While sufficiently high affinity is necessary for high processivity and thus efficient hydrolysis (no time is wasted by repeated enzyme detachment and rebinding), too high affinity may not only increase the processivity but at the same time prevent detachment and reuse of inactively bound enzyme molecules. This would point to an existence of optimum affinity, where the steady‐state hydrolysis rate reaches its maximum.

Several experiments have confirmed this link between the affinity and the steady‐state hydrolysis rate. For example, a W38A mutant of Cel7A exhibits a lower substrate affinity and lower processivity but a higher steady‐state hydrolysis rate (Kari et al., 2014). Similar effect has been reported in other studies (Nakamura et al., 2014; Sørensen et al., 2017), and also for another enzyme, the chitinase (Horn et al., 2006).

This behavior also follows directly from the proposed model, as explained in this section. The enzyme affinity for the substrate is determined by the attachment and detachment rate constants, the overall hydrolysis rate by the amount of the bound enzyme in the active state EC. In the proposed model, there are two types of detachment rate constants: the rate constant for the active enzyme EC detachment from the substrate ($k_{2}$), and the rate constant of leaving the blocked state ${\left(\text{EC}\right)}_{\text{b}}$ ($k_{4}$ in the model Ab and $k_{5}$ in model Aa). These rates have an opposite effect on the steady‐state level of the active species EC (see Equations (7) and (8)): higher concentration of EC, and therefore higher hydrolysis rate, can be achieved either by decreasing the rate constant $k_{2}$ or by increasing the rate constant $k_{4}$ (or $k_{5}$).

**Figure 8:**
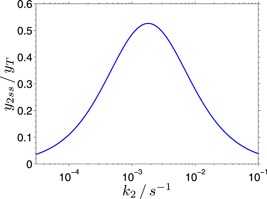
The maximum of hydrolytic activity for correlated rate constants $k_{2}$ and $k_{4}$. The hydrolytic activity is proportional to the steady‐state concentration $y_{2ss}$ of the active state EC. The following rate constants in the model Ab were used: $k_{4}=k_{2}$, $k_{1}=0.004\;s^{-1}$ (this corresponds approximately to the substrate concentration 11.2 μM), and $k_{3}=0.00081\;s^{-1}$ [Color figure can be viewed at wileyonlinelibrary.com]

Although these two rate constants are in general different from each other, it is reasonable to assume that they are not independent from each other, as they likely involve breaking of similar interactions (unthreading of the cellulose chain, or, less specifically, disrupting hydrophobic Trp‐pyranose interactions). Any modification of the enzyme (mutation) is, therefore, likely to change both rate constants in the same way: both will either increase or decrease. This relationship can be modeled by assuming that the two rate constants change proportionally with any enzyme modification, that is, their values will remain in a fixed relation: $k_{4}=ak_{2}$ (or $k_{5}=ak_{2}$). It can then be easily shown (from Equations (7) and (8)) that under this assumption there is an optimal rate $k_{2}=\sqrt{k_{1}k_{3}∕a}$ for which the steady‐state concentration of the active enzyme EC, and therefore the hydrolytic activity, reaches its maximum (Figure 8). Lower $k_{2}$ than this optimum decreases the concentration of EC in favor of the blocked state ${\left(\text{EC}\right)}_{\text{b}}$, higher $k_{2}$ leads to higher concentration of unbound enzyme E. The proposed model thus offers an explanation for the experimentally observed lower processivity and lower substrate affinity accompanied by a higher steady‐state hydrolysis rate.

### 4.4 Glucose production

**Figure 9:**
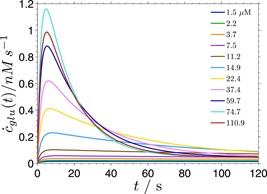
The glucose production rate derived from the proposed model Ab. The glucose production rate curves are calculated from the parameters of the fits shown in Figure 5 using the expression ${\dot{c}}_{glu}\left(t\right)=k_{7}y_{2}^{\prime }\left(t\right)$, where $y_{2}^{\prime }\left(t\right)$ is the concentration of the actively bound enzyme before performing the first cut in the processive run [Color figure can be viewed at wileyonlinelibrary.com]

Using the parameters of the fits to model Ab shown in Figure 5 and the solution of the modified model that includes glucose production in the first cut in the processive run (Figure 4), it is possible to calculate the time‐dependent production rate of glucose (Figure 9). Similarly to the overall hydrolysis rate, the glucose production rate exhibits an initial burst at higher substrate concentrations. This burst, however, peaks at very short times (between 5 and 15 s in the case here), and the glucose production rate reaches a quasi‐steady state much faster than the overall hydrolysis rate. In the data shown in Figures 5 and 9, this time coincides approximately with the maximum of the overall hydrolysis rate, several hundreds of seconds from the start of the experiment.

At lower substrate concentrations, the glucose production rate reaches the steady state also within a few seconds, but without a clear maximum. This kind of time‐dependent evolution of cellobiose and glucose production rates has been reported for TrCel7B (Murphy et al., 2012). Although this enzyme is classified as endoglucanase, it has been shown to exhibit a certain degree of processivity.

The glucose in this model is produced nonprocessively, since at most one glucose molecule is produced after the enzyme molecule reaches the active state. Nevertheless, a burst of glucose production can appear after the mixing of the free enzyme and the substrate at a sufficiently high substrate concentration (Figure 9). This shows that in the proposed model processivity is not the essential property needed for the appearance of the hydrolysis burst. The origin of the burst can be attributed to the existence of a third, inactive, state (here ${\left(\text{EC}\right)}_{\text{b}}$) that becomes populated slower than the active state (EC), regardless of whether the enzymatic hydrolysis is processive or not.

### 4.5 Second activity burst upon enzyme addition

**Figure 10:**
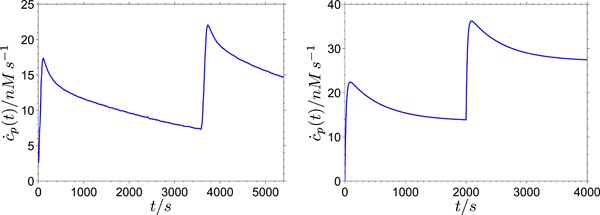
The second activity burst upon enzyme addition as observed experimentally (Praestgaard et al., 2011; left) and as predicted by model Ab (right) [Color figure can be viewed at wileyonlinelibrary.com]

It has been shown several times that addition of enzyme to the reaction mixture at a later time, after the initial hydrolysis burst has decayed, leads to a similar activity burst (Cruys‐Bagger et al., 2012; Eriksson, Karlsson, & Tjerneld, 2002; Murphy, Borch, McFarland, Bohlin, & Westh, 2010, 2012; Praestgaard et al., 2011). This has been interpreted as an evidence that the initial decrease of enzyme activity is not primarily related to the changes of the substrate (structure, reactivity, or density of accessible binding sites) but to the amount of the active enzyme (Yang et al., 2006). As the hydrolysis progresses, the amount of the active enzyme decreases at the expense of reversibly or permanently inactivated enzyme. The proposed model predicts an activity burst qualitatively similar to that observed experimentally (Figure 10). This means that the existence of the nonproductive bound state ${\left(\text{EC}\right)}_{\text{b}}$ is sufficient for the emergence of the second activity burst after enzyme addition, without the need to assume irreversible enzyme inactivation.

## 5 CONCLUSION

The proposed model of the initial cellulose hydrolysis achieves an equally good (Figure 6) or a much better (Figure 5, Supporting Information Figure S2) agreement with the experimental data than the published model, while maintaining a similar complexity. Notably, the new model eliminates the two shortcomings of the previous model by implicitly incorporating a nonconstant maximum length of a processive hydrolysis run. It should be stressed that the good match of the model with the data is not invoked by artificially including additional assumptions, such as irreversible enzyme inactivation. The simple analytical solution of the model is a major advantage for both the theoretical analysis of the model properties and the practical use of the model in fitting experimental data.

The time‐resolved model is complemented by a steady‐state theory, which is useful to describe the pseudo‐steady‐state reached some time after the initial activity burst. Importantly, the analysis of the expressions describing the steady state reveals that an optimal enzyme‐substrate affinity exists, at which the pseudo‐steady‐state rate of hydrolysis is maximized. The model thus provides theoretical basis for the previous experimental findings that fine‐tuning of the enzyme association and dissociation rates by enzyme engineering can improve the performance of cellulases, despite decreasing their substrate affinity and their processivity (Kari et al., 2014).

Interestingly, the modeling of the glucose production rate in the first hydrolysis step indicates that in the proposed model processivity is not a necessary prerequisite for the appearance of the activity burst. We furthermore showed that the proposed model reproduces the appearance of a second activity burst following the addition of more enzyme.

The proposed model reproduces several different aspects of hydrolysis by *Tr*Cel7A that were previously observed experimentally, and therefore has the potential to deepen the general understanding of processive hydrolysis by cellulases. The central limiting factor appears to be the blocked state. Its existence is supported by a range of previous studies (Igarashi et al., 2011; Jalak & Väljamäe, 2010; Kurašin & Väljamäe, 2011; Väljamäe et al., 1998; Yang et al., 2006). Answering the questions regarding its nature, the possibility of its reduction or elimination, its dependence on the substrate origin and pretreatment, etc., will, therefore, open new ways for enhancing cellulose hydrolysis.

The presented results are furthermore expected to contribute to our understanding of the kinetics of other processive hydrolytic enzymes, such as chitinases and amylases, and, more generally, other enzymes processively interacting with biopolymers, for example, processive proteases (Pickart & Cohen, 2004), DNA motor enzymes, and motor proteins processively translocating along cytoskeletal filaments (Kinbara & Aida, 2005).

## Supporting information

Supplementary Information
