# Supplementary material for: Modeling the activity burst in the initial phase of cellulose hydrolysis by the processive cellobiohydrolase Cel7A
Source: Biotechnol Bioeng. 2019 Jan 8;116(3):515–25. doi: 10.1002/bit.26889 (PMC6590443; doi:10.1002/bit.26889)
Supplement: Supplementary file 1 — Supplementary Information [file BIT-116-515-s001.pdf]

Supplementary Information

The full model and its simplified versions

In order to construct a minimal model of the initial phase of cellulose hydrolysis by processive cellulase, we make several simplifying assumptions. As we are interested only in the initial phase, before a significant fraction of the substrate is hydrolyzed, we assume that the substrate concentration, or more precisely, the concentration of enzyme binding sites on the substrate, remains constant. It is also assumed, that the binding sites on the substrate are far from saturated by the available enzyme. This means that the enzyme binding rate is constant, independent of the concentration of already bound enzyme. Finally, we neglect any long-time effects, such as permanent enzyme inactivation, which might otherwise play a role in a prolonged hydrolysis experiment.

A model with two enzyme states (a free, unbound and an active, substrate-bound state) and with transitions between these states described by simple rates ($1 \rightleftharpoons 2$) cannot result in the observed maximum of activity. Such a model exhibits an exponential approach to equilibrium without any maxima. Therefore, a third state has to be included. This state, following experimental evidence, can be interpreted as a non-productive substrate-bound enzyme. It can be an enzyme blocked by an obstacle, with a threaded cellulose chain or without it, or an non-productively bound enzyme diffusively searching for a cellulose chain end on the substrate surface, etc.

With these 3 states, 6 rate constants describe the conversion between the states (Fig. 2). Given the usually limited experimental data, these six rates, together with the actual rate of hydrolysis, are too many parameters to determine experimentally. But perhaps some of the rates are less significant, and can be set to zero in a simplified model. What is the minimum number of rates that need to be considered to fit the data? At least three processes are necessary: binding to the substrate, release from the substrate, and transition from one bound state to the other (either directly or via detachment from the substrate). Furthermore, it is sufficient to consider only models in which the free enzyme in solution does not become fully depleted (that is, detachment from the substrate is allowed). The only configurations with only three rates that avoid depletion of the

unbound state are cyclic arrangements between the states: E $\rightarrow$ EC $\rightarrow$ $(EC)_b$ $\rightarrow$ E or E $\rightarrow$ $(EC)_b$ $\rightarrow$ EC $\rightarrow$ E. These configurations are rather restrictive, and when used to fit the experimental data did not lead to good data fits. It is therefore worth considering models with four rates.

There are in total 15 different combinations of four non-zero rates, six of which can be directly excluded because they do not allow either attachment to or detachment from the substrate or access to one of the two bound states, or they lead to the accumulation of all enzyme in one state. The remaining nine four-rate models can be categorized into three classes (A, B, C), each containing 3 models, depending on the state in which the enzyme is upon binding to the substrate (Fig. S1). The enzyme can bind to be immediately active (EC), with the blocked state $(EC)_b$ entered later (class A), or the enzyme can bind to become either active or blocked (class B), or the enzyme binds to be in the blocked state $(EC)_b$ (which could be interpreted as, for example, a state searching for the active site, that is, the cellulose chain end), only to enter the active (hydrolyzing) state later (class C).

A detailed analysis of the four-rate models in classes A, B and C shows that the models in class C cannot produce an activity maximum similar to the one observed experimentally. All six models in classes A and B can produce a clear activity burst for some range of kinetic parameters. The quality of fits to the experimental data varies among the models; the choice of a 'favourable' model is however complicated by the equivalence of some models, as described in more detail in the following section.

Model equivalence

A closer inspection of the four-rate models reveals that the solutions of some of these are mathematically equivalent to each other, with respect to the type of data used in this article. By equivalence we mean here that one model can be converted into another by transforming the set of rates k_i of one model to the set of rates k'_i of the other model while preserving Eq. 2, which defines the problem and its solution. As a result, the solutions of the two models, $y_i(t)$ and $y'_i(t)$ are equal, up to a scaling factor. A direct consequence of this fact is that one cannot distinguish between these

models when describing the experimental data. An exception to this indistinguishability is the situation when one of the two equivalent models can be excluded because it contains non-physical parameter values, for example, negative rate constants.

A search for parameter transformations among the nine models in Fig. S1 preserving the form of Eq. 2 shows that there are three groups of equivalent models. These are described in more detail below. In all cases we set $y_T = 1$ for simplicity and without a loss of generality; the concentrations y_1 and y_2 have then the meaning of fractions of enzyme molecules in the two states. The concentrations of the free species $y_1(t)$ are equal in both models: $y_1'(t) = y_1(t)$ because of the initial condition $y_1(0) = y_1'(0) = 1$ (all enzyme molecules are free at the beginning of the experiment). The concentrations of the bound active species EC can be related by a scaling factor β: $y_2'(t) = \beta y_2(t)$. This scaling degree of freedom is present because experimentally we do not obtain the absolute concentration $y_2(t)$ but only the rate of cellobiose formation, which is proportional to $y_2(t)$. In order to eliminate this model equivalence, more experimental data would be necessary. The three groups of equivalent models are: (Ab, Ba), (Aa, Bb, Bc) and (Ca, Cb). In the following, several examples of transformations between equivalent models are given.

Ab — Ba

The model Ab can be converted into the model Ba by the following transformation (the parameters of Ba are marked with a prime):

$$\beta = \frac{1}{2k_4}\left(k_2 + k_3 + k_4 - \sqrt{(k_2 + k_3 + k_4)^2 - 4k_2k_4}\right), \tag{S1}$$

$$k_1' = \beta k_1, \qquad k_2' = k_2/\beta, \qquad k_4' = \beta k_4, \qquad k_6' = (1-\beta)k_1. \tag{S2}$$

This means that if $y_1(t)$ and $y_2(t)$ are the solutions of the model Ab (Eq. 2) with the parameters k_1, k_2, k_3, k_4, then $y_1'(t) = y_1(t)$ and $y_2'(t) = \beta y_2(t)$ are the solutions of the model Ba with the parameters k_1', k_2', k_4', k_6'. As long as both sets of parameters are physically meaningful, we cannot

distinguish between the two models based on the unscaled $y_2(t)$ data alone (the experimentally determined time evolution of the rate of cellobiose production). Similar reasoning applies to the following model groups.

Aa — Bb — Bc

The model Aa can be converted into the model Bb by the following transformation (the parameters of Bb are marked with a prime):

$$\beta = \frac{k_2 - k_5}{k_2 - k_5 + k_3}, \tag{S3}$$

$$k_1' = \beta k_1, \qquad k_2' = k_2 + k_3, \qquad k_5' = k_5, \qquad k_6' = (1-\beta)k_1. \tag{S4}$$

The transformation is physically meaningful only if $k_2 > k_5$ because β must be positive. The model Aa can be converted into the model Bc by the following transformation (the parameters of Bc are marked with a prime):

$$\beta = 1 - \frac{k_2}{k_5}, \tag{S5}$$

$$k_1' = \beta k_1, \qquad k_3' = k_2 + k_3, \qquad k_5' = k_5, \qquad k_6' = (1-\beta)k_1. \tag{S6}$$

Again, the transformation is physically meaningful only if $k_2 < k_5$ because β must be positive. This condition is complementary to the condition for the transformation Aa $\rightarrow$ Bb shown above; the model Aa is therefore meaningfully equivalent to either Bb or Bc but not to both models at the same time.

The model Bb can be converted into the model Bc by the following transformation (the parameters of Bc are marked with a prime):

$$\beta = 1 - \frac{k_2}{k_5}, \tag{S7}$$

$$k_1' = \beta k_1, \qquad k_3' = k_2, \qquad k_5' = k_5, \qquad k_6' = k_6 + (1-\beta)k_1. \tag{S8}$$

The transformation is physically meaningful only if $k_2 < k_5$ because β must be positive.

Ca — Cb

The model Ca can be converted into the model Cb by the following transformation (the parameters of Cb are marked with a prime):

$$\beta = 1 - \frac{k_2}{k_5}, \tag{S9}$$

$$k_3' = k_2 \left(1 + \frac{k_4}{k_5}\right), \qquad k_4' = \beta k_4, \qquad k_5' = k_5, \qquad k_6' = k_6. \tag{S10}$$

The transformation is physically meaningful only if $k_2 < k_5$ because β must be positive.

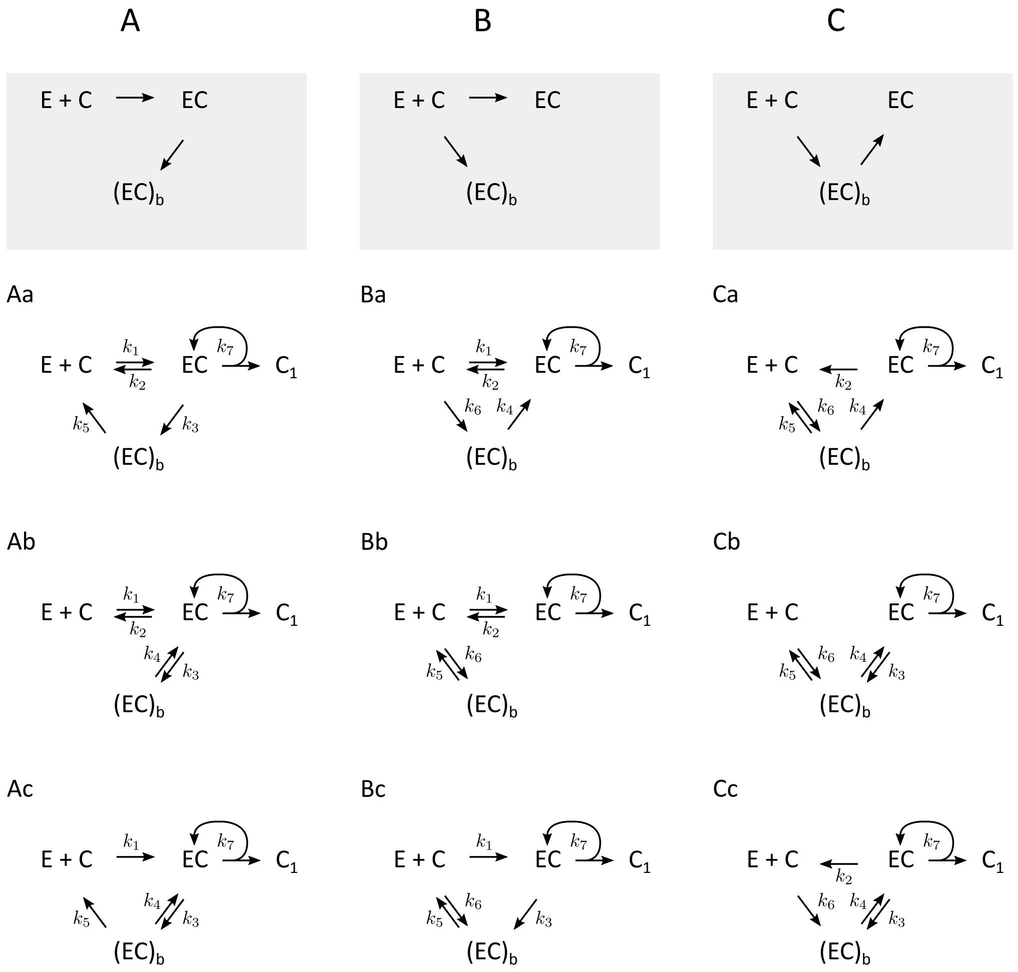

Figure S1: The four-rate models divided into three classes: A, B and C. In the first row, the defining characteristics of the three classes — how the bound enzyme states EC and $(\mathrm{EC})_\mathrm{b}$ are reached from the unbound state E — are shown. The arrows in the first row depict only the order in which the two bound states are populated, and do not represent all possible reactions. The nine models in the remaining rows are shown with all reaction rates: the four rate constants k_i describing the change of the state of the enzyme, and the rate constant k_7 of the production of cellobiose.

Processivity

We use the definition of processivity as the average number $\overline{n}$ of hydrolysis steps during one processive run, that is, while the enzyme is in the active bound state EC (intrinsic processivity):

$$\overline{n} = \sum_{i=0}^{\infty} i p_i, \tag{S11}$$

where p_i is the probability, that i units will be hydrolyzed during one processive run. The probability p_i depends on the duration t of the processive run and can be calculated by integrating over all possible durations of the processive run:

$$p_i = \int_{t=0}^{\infty} p(i,t) p_T(t)\, dt, \tag{S12}$$

where $p(i,t)$ is the probability of i hydrolysis steps during a processive run of length t, and $p_T(t)$ is the probability density that the processive run has a duration t. The probability $p(i,t)$ is a Poisson distribution of i with the mean value $k_7 t$, where k_7 is the rate constant of hydrolysis:

$$p(i,t) = e^{-k_7 t} \frac{(k_7 t)^i}{i!}. \tag{S13}$$

The probability $p_T(t)dt$ of leaving the active bound state EC after time t can be expressed as the product of the probability that the enzyme does not leave the EC state during the time interval $(0,t)$: $e^{-k_2 t}$ and the probability that it leaves the state EC during the interval $(t, t+dt)$: $k_2 dt$, where k_2 is the rate constant of leaving the EC state:

$$p_T(t)dt = e^{-k_2 t} k_2\, dt. \tag{S14}$$

Combination of the above equations yields:

$$\overline{n} = \sum_{i=0}^{\infty} i \int_{t=0}^{\infty} e^{-k_7 t} \frac{(k_7 t)^i}{i!}\, e^{-k_2 t} k_2\, dt = \int_{t=0}^{\infty} k_7 t\, e^{-k_2 t} k_2\, dt = \frac{k_7}{k_2}. \tag{S15}$$

In the models Aa and Ab, where the active bound state EC can be left either by detachment (rate constant k_2) or by entering the blocked state (rate constant k_3), k_2 in eq. S14 is replaced by the sum of these two rate constants $k_2 + k_3$, resulting in the processivity $\overline{n} = k_7/(k_2 + k_3)$.

Using the above definition, the processivity in the published model (Praestgaard et al., 2011) would be k_2/k_3 (meaning $k_{\mathrm{cat}}/k_{\mathrm{off}}$) if the chain length were not restricted to $n = 150$ units. With this restriction, the processivity is $\overline{n} = k_2/k_3(1 - (k_2/(k_2 + k_3))^n)$.

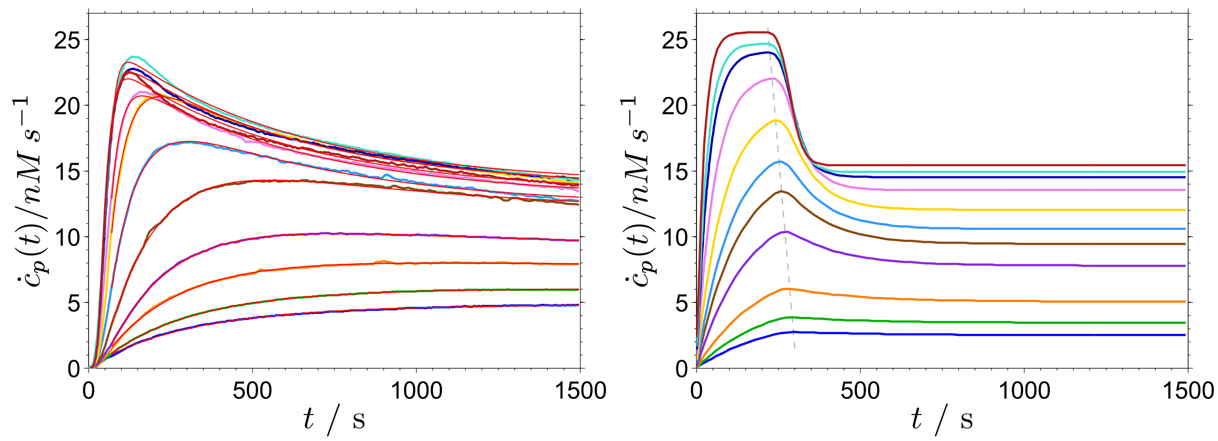

Figure S2: Comparison of fits of the proposed model Ab and the published model to the published data (Praestgaard et al., 2011). The different curves correspond to initial substrate concentrations varied from 1.5 μM (the lowest curve) to 110.9 μM; left: the data and the fits of the Ab model (red lines); right: the fits of the published model. In both cases all fit curves in one plot share the same rate constants k_i (global fit). Note the abrupt decrease of the production rate in the published model at approx. 270 s, and the fast relaxation to a constant value, both absent in the data.

Experimental details

The data fitted in Fig. 5 and S2 were taken from ref. (Praestgaard et al., 2011). The enzyme concentration in all measurements was 50 nM. The substrate was reconstituted amorphous cellulose (RAC) prepared from cellulose Sigmacell 20. The substrate concentrations, expressed as the concentration of reducing ends, ranged from 1.5 μM to 110.9 μM. The hydrolytic activity of the enzyme was measured by a calorimetric method; the cellobiose produced by hydrolysis of cellulose was further converted to glucose and D-glucono-δ-lactone by added β-glucosidase and glucose oxidase, thus siginificantly amplifying the heat signal. The measured heat flow (in $J{\cdot}s^{-1}$) was converted to the rate of cellobiose production. The response time of the calorimeter was approximately 15 s. The measurements were performed at 25℃. For more details see the original publication (Praestgaard et al., 2011).

The data fitted in Fig. 6 were taken from ref. (Cruys-Bagger et al., 2012). The enzyme concentration was 50 nM. The substrate was reconstituted amorphous cellulose (RAC) prepared from cellulose Sigmacell 20. The substrate concentrations were between 0.25 and 2.0 $g{\cdot}l^{-1}$. The concentration of cellobiose produced by cellulose hydrolysis was measured by an electrochemical biosensor based on the detection of the oxidation current produced by cellobiose dehydrogenase immobilized on the biosensor electrode. The time constant of the biosensor was approximately 1 s. The measurements were performed at 25℃. For more details see the original publication (Cruys-Bagger et al., 2012).

References

Cruys-Bagger, N., Elmerdahl, J., Praestgaard, E., Tatsumi, H., Spodsberg, N., Borch, K., & Westh, P. (2012, May). Pre-steady-state kinetics for hydrolysis of insoluble cellulose by cellobiohydrolase Cel7A. *J. Biol. Chem.*, *287*(22), 18451–18458. doi: 10.1074/jbc.M111.334946

Praestgaard, E., Elmerdahl, J., Murphy, L., Nymand, S., McFarland, K. C., Borch, K., & Westh, P. (2011, May). A kinetic model for the burst phase of processive cellulases. *FEBS J.*, *278*(9), 1547–1560. doi: 10.1111/j.1742-4658.2011.08078.x
